# Hydroxyapatite supported caesium carbonate as a new recyclable solid base catalyst for the Knoevenagel condensation in water

**DOI:** 10.3762/bjoc.5.68

**Published:** 2009-11-20

**Authors:** Monika Gupta, Rajive Gupta, Medha Anand

**Affiliations:** 1 Department of Chemistry, University of Jammu, Jammu 180 006, India

**Keywords:** ethyl cyanoacetate, hydroxyapatite supported caesium carbonate, Knoevenagel condensation, malonic acid, malononitrile

## Abstract

The Knoevenagel condensation between aromatic aldehydes and malononitrile, ethyl cyanoacetate or malonic acid with hydroxyapatite supported caesium carbonate in water is described. HAP–Cs_2_CO_3_ was found to be a highly active, stable and recyclable catalyst under the reaction conditions.

## Introduction

An area of recent intense synthetic endeavour is the use of approaches that are beneficial to industry as well as to the environment. Since Green Chemistry is primarily concerned with the reduction of chemical hazards and pollution [1], a plausible convention is to use safer solvents that pose considerably less threat to ecosystems. Thus, the use of aqueous solvents in chemical reactions has proved a cleaner and safer alternative to organic solvents [2].

Heterogeneous catalysis, which has the immediate advantage of easy recoverability and recyclability of the catalyst – sometimes with the further advantage of increased selectivity of the desired product over traditional homogeneous systems – is a pivotal process in organic synthesis. Diverse ranges of heterogeneous solid supports have been employed so far. Recently, hydroxyapatite has proved to be a highly efficient catalyst due to its promising ability as a macro-ligand for catalytically active centres [3–5]. Hydroxyapatites can be used as biomaterials, adsorbents, ion-exchangers and catalysts. So far, a few excellent applications of hydroxyapatites as catalysts or catalytic supports have emerged [6].

Solid base catalysts are known to display unique catalytic performance across a diverse array of C–C bond forming reactions. Without doubt, an alternative strategy for the Knoevenagel condensation [7] by this approach is worthwhile since it produces an extended domain of useful products for use in the pharmaceutical and biomedical industries. Arylidenemalononitriles are primarily used in the synthesis of fine chemicals in agriculture and medicine and as precursors of heterocycles with biological activity [8–16] whilst arylidene derivatives of ethyl cyanoacetate are successfully employed in the synthesis of cyanocoumarins [17–18], as antimetabolites [19] and in particular as synthetic intermediates for many heterocyclic compounds [20]. Moreover, cinnamic acid derivatives have major applications in many fields and find applications as plasticizers, perfumes, aroma compounds [21], lubricants, etc. The diverse range of catalysts known to effect the Knoevenagel condensation includes modified hydrotalcites [22], amines [23–24], K_2_CO_3_ [25], Lewis acid catalysts [26–28] and ionic liquids [29]. There is a variety of procedures available for carrying out Knoevenagel condensation in green solvents. The Knoevenagel condensation is strongly solvent-dependent [30–31]. In addition, the use of aqueous and highly protic solvents is currently of great importance since it avoids the problems of self-condensation, 1,2-elimination and retro-Knoevenagel condensation reactions.

Herein, we report the facile preparation of hydroxyapatite supported caesium carbonate (HAP–Cs_2_CO_3_) and its effective application to the Knoevenagel condensation between different aromatic aldehydes and malononitrile or ethyl cyanoacetate or malonic acid by stirring in water at 80–100 °C. The products were obtained in high yield and purity.

## Results and Discussion

### Preparation and characterization of HAP–Cs_2_CO_3_

Hydroxyapatite needed for the preparation of hydroxyapatite supported caesium carbonate catalyst was obtained by the co-precipitation method with slight procedural changes [32–37]. HAP–Cs_2_CO_3_ was prepared by stirring the mixture of hydroxyapatite and Cs_2_CO_3_ in double distilled water (DDW) for 4 h. The solid was filtered, washed with DDW and dried overnight first at 100 °C (373 K) and later at 700–800 °C in a muffle furnace for 1 h.

### Characterization of HAP–Cs_2_CO_3_

**Figure 1:**
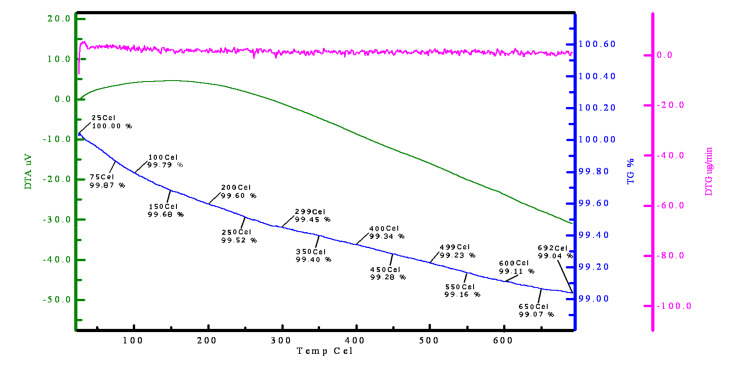
TGA of HAP–Cs_2_CO_3_.

**Figure 2:**
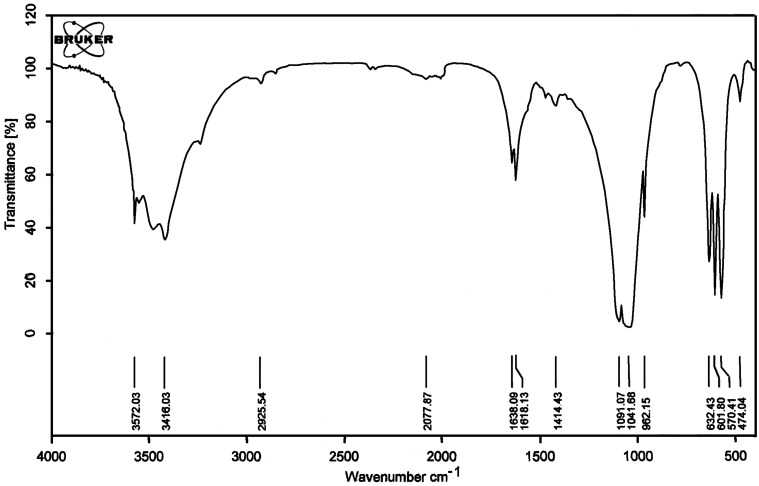
FTIR spectrum of HAP–Cs_2_CO_3._

The characterization of HAP–Cs_2_CO_3_ was done by Fourier transform spectroscopy (FTIR) and thermogravimetric analysis (TGA, Figure 1). The FTIR of HAP–Cs_2_CO_3_ showed a strong absorption band at 3572 cm^−1^ attributed to the presence of OH^−^ ions in a calcium hydroxyapatite lattice. The presence of CO_3_^2−^ ion was confirmed by a weak absorption band at 1615 cm^−1^ (Figure 2). The TGA curve showed very little weight loss up to 692 °C (0.96 g), which may be attributed to the loss of organic solvent or water molecules trapped on the surface of hydroxyapatite. The TGA data indicated the high stability of HAP–Cs_2_CO_3_ up to 692 °C (Figure 1). It has not yet proved possible to determine the Cs content of this catalyst.

### The Knoevenagel condensation reaction

Knoevenagel condensation between aromatic aldehydes and malononitrile or ethyl cyanoacetate in the presence of catalyst HAP–Cs_2_CO_3_ in DDW as solvent was investigated. In order to optimize the reaction conditions for the Knoevenagel condensation, a different set of reactions was carried out with respect to different molar ratios of HAP–Cs_2_CO_3_, temperature, substrates and solvent. 4-Chlorobenzaldehyde and malononitrile were selected as the test substrates. It was found that no reaction occurred between 4-chlorobenzaldehyde and malononitrile in DDW at 80 °C. The same reaction was carried out with HAP. After 15 h TLC indicated that the reaction had proceeded very slightly and, after work up, the product was isolated in only 2% yield. Similar results were obtained when the reaction was repeated with Cs_2_CO_3_ (reaction time 22 h) and gave the isolated product in 5% yield. Subsequent to these investigations, one further experiment needed to be carried out: to pre-saturate the aqueous phase with caesium carbonate and then decant off the aqueous portion. The aqueous portion was then used in a similar experiment as outlined above but gave only a 4–5% yield of product, consistent with the poor solubility of caesium carbonate in water. As a consequence, we surmised that reaction needed to be carried out with HAP–Cs_2_CO_3_. The amount of HAP–Cs_2_CO_3_ was optimized with the test substrates and with 0.1–0.5 g of the catalyst. It was found that 0.3 g of catalyst gave optimum results in terms of reaction time and yield. Secondly, the reaction with the test substrates was carried out at different temperatures (40, 60, 80 and 100 °C) and 80 °C was found to be the optimum reaction temperature. A comparative study was carried out with calcium carbonate. This appears to be a better catalyst but suffers from the disadvantages of both poor solubility in water and a low reaction rate. Recently, more emphasis has been laid on replacing toxic solvents with non-polluting green solvents such as water. Therefore we carried out the reaction with DDW as solvent. The amount of water as solvent was optimized with 5–10 mL of DDW. It was found that 7 mL of water was sufficient to carry out the reaction in an efficient, mild and cost-effective way. Thus, the optimum conditions were established as: aldehyde (1 mmol), malononitrile (1 mmol), HAP–Cs_2_CO_3_ (0.3 g) and water (7 mL) at an optimum temperature of 80 °C. Under these conditions, product **3c** (Table 1) was obtained in 86% isolated yield after 2 h.

**Table 1:** HAP–Cs_2_CO_3_ catalyzed Knoevenagel condensation between aromatic aldehydes and malononitrile or ethyl cyanoacetate at 80–100 °C with water as solvent.

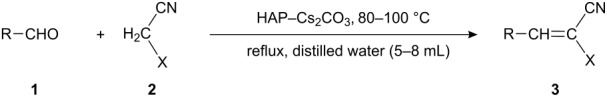					

Products^a^	R	X	Time (h)	Yield (%)^b^	mp (Lit. mp) °C

**3a**	C_6_H_5_	CN	2.5	77	80–81 (81–82) [21]
**3b**	4-OCH_3_C_6_H_4_	CN	3	74	114–115 (115–118) [21]
**3c**	4-ClC_6_H_4_	CN	2	86	159–162 (160–163) [21]
**3d**	4-OHC_6_H_4_	CN	3.5	68	188–189 (187–188) [21]
**3e**	3-NO_2_C_6_H_4_	CN	2.5	73	102–104 (103–104) [21]
**3f**	3-OCH_3_-4-OHC_6_H_3_	CN	2	72	135–137 (135–136) [21]
**3g**	2-OHC_6_H_4_	CN	1.5	75	98–99 (100–101) [21]
**3h**	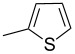	CN	2	77	102–104 (102–105) [21]
**3i**	–CH=CH–C_6_H_4_	CN	3	73	125–127 (126–129) [21]
**3j**	C_6_H_5_	COOEt	15	75	49–50 (49–52) [21]
**3k**	4-ClC_6_H_4_	COOEt	13	82	89–90 (90–94) [21]
**3l**	4-OHC_6_H_4_	COOEt	15	80	87–88 (88–90) [21]
**3m**	3-NO_2_C_6_H_4_	COOEt	17	78	128–130 (128–132) [21]
**3n**	4-CH_3_C_6_H_4_	COOEt	14	75	91–92 (90–92) [21]
**3o**	–CH=CH–C_6_H_5_	COOEt	17	71	112–114 (114–115) [21]

^a^All the products were characterised by ^1^H NMR, IR, mass spectral data and comparison with authentic samples available commercially or prepared according to the reported methods. ^b^Yields refer to the isolated yields.

To demonstrate the versatility of the developed protocol, different aldehydes – substituted with both electron-withdrawing and electron-releasing groups – were chosen and excellent results were obtained (Table 1). This methodology was also applicable to heteroaromatic aldehydes (**3h**, Table 1) and unsaturated aldehydes (**3i**, Table 1). Promising results were obtained and the corresponding products were isolated in 77 and 73% yields after 2 and 3 h, respectively.

There is no data with respect to the rate of condensation versus dehydration or 1,2-elimination of the product. In all cases, no doubt, the yields were appreciable, but involved tedious separation. Electrochemically induced Knoevenagel condensations [38] have been reported but require solvents with particular characteristics (suitable potential range, high dielectric constant, polarity, etc.) and are, in addition, less competitive than classical organic syntheses.

The condensation between aromatic aldehydes and ethyl cyanoacetate showed higher selectivity than malononitrile with aldehydes with electron-withdrawing and electron-releasing groups (Table 1), under optimum reaction conditions with respect to molar ratio of substrate, catalyst (HAP–Cs_2_CO_3_), reaction temperature and amount of solvent.

In all cases, good yields were observed as shown in Table 1. A test reaction was carried out between 4-chlorobenzaldehyde (1 mmol) and ethyl cyanoacetate (1 mmol), HAP–Cs_2_CO_3_ (0.3 g) at a reaction temperature of 100 °C in DDW (7 mL). It was found that the product was obtained in 82% isolated yield after 13 h (**3k**, Table 1).

Reactions with our catalyst with water as the solvent in a 80–100 °C temperature range gave products in excellent yields e.g. 86% in the case of (4-chlorobenzylidene)malononitrile (**3c**, Table 1).

Knoevenagel condensation between aromatic aldehydes and malonic acid was also investigated with a slight modification in the amount of catalyst used i.e. 0.2 g of HAP–Cs_2_CO_3_ at a temperature of 80 °C with water as solvent. Excellent results were obtained with this mild procedure. A variety of aromatic aldehydes with electron-withdrawing and electron-releasing groups were chosen to investigate the generality of this methodology (Table 2).

**Table 2:** HAP–Cs_2_CO_3_ catalysed Knoevenagel condensation between aromatic aldehydes and malonic acid at 80 °C using water as solvent.

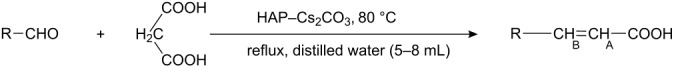				

Products^a^	R	Time (h)	Yield (%)^b^	mp (Lit. mp) °C

**3a**	C_6_H_5_	4	76	136–137 (135–136)
**3b**	4-ClC_6_H_4_	3	88	245–247 (248)
**3c**	4-OCH_3_C_6_H_4_	4.5	80	198–200 (200–202)
**3d**	2-OHC_6_H_4_	3.5	79	215–217 (216–218)
**3e**	3-OCH_3_-4-OHC_6_H_3_	4	76	287–289 (286)
**3f**	3-NO_2_C_6_H_4_	4.5	75	199–200 (200–202)
**3g**	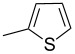	3	76	153–154 (152–154)
**3h**	3,4-(OCH_3_)_2_C_6_H_3_	4	85	182–183 (182–184)
**3i**	4-OHC_6_H_4_	6	71	213–214 (214)

^a^All the products were characterised by ^1^H NMR, IR, mass spectral data and comparison with authentic samples available commercially or prepared according to the reported methods. ^b^Yields refer to the isolated yields.

The yield of 3-(4-chlorophenyl)prop-2-enoic acid with this mild procedure was 88% (**3b**, Table 2).

The protocol described above provides an efficient method for Knoevenagel condensation between aromatic aldehydes and active methylene groups using HAP–Cs_2_CO_3_ as a new recyclable solid base catalyst in water. Moreover, the recyclability of this catalyst (HAP–Cs_2_CO_3_) makes the method very cost-effective.

### Heterogeneity and recyclability of the catalyst

When using supported heterogeneous catalysts, there are two important points to consider: heterogeneity and recyclability. To rule out the possibility of homogeneous catalysis, a reaction between 4-chlorobenzaldehyde and malononitrile was carried out in the presence of HAP–Cs_2_CO_3_, with water as solvent, for 30 min. After this time the catalyst was filtered off and the filtrate was used for a reaction under similar conditions. It was found that very little conversion was observed. This result clearly indicates that the catalysis is purely heterogeneous. Similarly, the reaction between 4-methoxybenzaldehyde and malonic acid was also carried out to investigate heterogeneity. The second point is the recyclability and deactivation of the heterogeneous catalyst which are equally important when supported catalysts are employed. This was accomplished by carrying out the reactions between 4-chlorobenzaldehyde and malononitrile, and additionally, the reaction between 4-methoxybenzaldehyde and malonic acid for five consecutive runs with the same catalyst as shown in Table 3. After every use, a very little loss of catalytic activity was observed which may be attributed to the microscopic changes in the structure of the catalyst.

**Table 3:** Recyclability of HAP–Cs_2_CO_3_ as demonstrated for 4-methoxybenzaldehyde and malonic acid in water at 80 °C.

No. of runs^a^	Time (h)	Yield (%)^b^

1	4.5	80
2	5	78
3	6	78
4	7.5	75
5	9	72

^a^No. of runs. ^b^Yields refer to the isolated yields.

## Conclusion

An efficient Knoevenagel condensation was achieved between a range of aromatic aldehydes and active methylene compounds in short time periods by using HAP–Cs_2_CO_3_ as the catalyst and water as the solvent under mild conditions. Due to its reusability, this catalyst is an attractive candidate for commercial realisation of C–C bond forming reactions. Moreover, this method is an environmentally safer alternative to existing methods.

## Experimental

### General

The melting points were taken in a Perfit Melting Point apparatus and are uncorrected. All products were identified by comparison of analytical data (melting point, IR and ^1^H NMR) with those of reported authentic samples. IR spectra were recorded as KBr discs on an ESI-Esquire 3000 Bruker Daltonics Spectrometer. ^1^H NMR spectra were recorded on a Bruker DPX 200 (200 MHz) spectrometer in CDCl_3_ solution with TMS as internal standard. The purity of the compounds was checked by thin layer chromatography on silica gel G and visualisation with iodine vapour or 2,4-dinitrophenylhydrazine spray. TGA was recorded on a Perkin Elmer Pyris Diamond Thermal Analyser in the temperature range 0–600 °C, with a heating rate of 10 K/s.

### Preparation of hydroxyapatite

To a solution (250 mL) containing diammonium hydrogenphosphate (7.92 g) (maintained at a pH greater than 12 by the addition of ammonium hydroxide, 60–70 mL) in a round-bottom flask, a solution (150 mL) containing calcium nitrate (23.6 g) was added. The reaction mixture was stirred for 4 h under reflux. DDW was used to prepare the solutions. The precipitated hydroxyapatite (HAP) was filtered off, washed with DDW, dried overnight at 80 °C (353 K) and calcined in air at 700 °C (973 K) for 30 min before use.

### Preparation of hydroxyapatite supported caesium carbonate (HAP–Cs_2_CO_3_)

To a mixture of hydroxyapatite (3 g) and Cs_2_CO_3_ (2.92 g) in a round-bottom flask, DDW (10–15 mL) was added. The reaction mixture was stirred for 4 h at room temperature. The solid was filtered, washed with DDW, dried overnight at 100 °C (373 K) and later at 700–800 °C in a muffle furnace for 1 h.

### A typical procedure for the Knoevenagel condensation between aromatic aldehydes and malononitrile or ethyl cyanoacetate in the presence of HAP–Cs_2_CO_3_ using water as solvent

To a mixture of aldehyde (1 mmol), malononitrile (1 mmol) or ethyl cyanoacetate (1 mmol) and distilled water (7 mL) in a round-bottom flask, HAP–Cs_2_CO_3_ (0.2 g for malononitrile or 0.3 g for ethyl cyanoacetate) was added. The reaction mixture was stirred at 80 °C (in case of malononitrile) or 100 °C (for ethyl cyanoacetate) in an oil-bath for the appropriate time. After completion of the reaction (monitored by TLC), the reaction mixture was diluted with ethyl acetate and the HAP–Cs_2_CO_3_ was filtered off. The organic layer was separated and dried over anhydrous Na_2_SO_4_. The product was obtained after removal of the solvent under reduced pressure and crystallisation from ethanol. The catalyst was washed with distilled water (20 mL) followed by ethyl acetate (15 mL) and dried 1 h at 100 °C for further use. The structures of the products were confirmed by IR, ^1^H NMR, mass spectral data and comparison with authentic samples.

### A typical procedure for the Knoevenagel condensation between aromatic aldehydes and malonic acid in the presence of HAP–Cs_2_CO_3_ using water as solvent

To a mixture of aldehyde (1 mmol), malonic acid (1 mmol) and distilled water (7 mL) in a round-bottom flask (50 mL), HAP–Cs_2_CO_3_ (0.2 g) was added. The reaction mixture was stirred at 80 °C in an oil-bath for the appropriate time. After completion of the reaction (monitored by TLC), the reaction mixture was diluted with ethyl acetate and the HAP–Cs_2_CO_3_ was filtered off. The organic layer was separated and dried over anhydrous Na_2_SO_4_. The product was obtained after removal of the solvent under reduced pressure and crystallisation from ethanol. The catalyst was washed with distilled water (20 mL) followed by ethyl acetate (15 mL) and dried 1 h at 100 °C for further use. The structures of the products were confirmed by IR, ^1^H NMR, mass spectral data and comparison with authentic samples.
